# Supplementary material for: Ancestral Sperm Ecotypes Reveal Multiple Invasions of a Non-Native Fish in Northern Europe
Source: Cells. 2021 Jul 9;10(7):1743. doi: 10.3390/cells10071743 (PMC8304145; doi:10.3390/cells10071743)
Supplement: Supplementary file 1 [file cells-10-01743-s001.zip › cells-1213104-SI.pdf]

Supplementary information for:

Ancestral Sperm Ecotypes Reveal Multiple Invasions of a Non-native Fish in Northern Europe

Leon Green[1,2,3,]*, Apostolos Apostolou[4], Ellika Faust[2,5], Kajsa Palmqvist[1], Jane W. Behrens[6], Jonathan N. Havenhand[2,5], Erica H. Leder[2,5,7] and Charlotta Kvarnemo[1,2]

1 Department of Biological and Environmental Sciences, University of Gothenburg, Medicinaregatan 18A, 41390 Gothenburg, Sweden; kajsa.palmqvist@gmail.com (K.P.); lotta.kvarnemo@bioenv.gu.se (C.K.)
2 Linnaeus Centre for Marine Evolutionary Biology, University of Gothenburg, Tjärnö Marine Laboratory, Laboratorievägen 10, 45296 Strömstad, Sweden; ellika.faust@marine.gu.se (E.F.) jon.havenhand@marine.gu.se (J.N.H.); erica.leder@marine.gu.se (E.L.)
3 Gothenburg Global Biodiversity Centre, University of Gothenburg, Karl Skottbergs gata 22, 41390 Gothenburg, Sweden
4 Institute for Biodiversity and Ecosystem Research, Bulgarian Academy of Sciences, Tsar Osvoboditel Blvd. 1, 1000 Sofia, Bulgaria; apostolosfish@abv.bg
5 Department of Marine Sciences, University of Gothenburg, Tjärnö Marine Laboratory, Laboratorievägen 10, 45296 Strömstad, Sweden
6 National Institute of Aquatic Resources, Technical University of Denmark, Kemitorvet, Building 202, 2800 Kgs. Lyngby, Denmark; jabeh@aqua.dtu.dk
7 Natural History Museum, University of Oslo, Sars' gate 1, 0562 Oslo, Norway
* Correspondence: leon.green@bioenv.gu.se

Supplementary table 1. Sites, their meta data, sources and summary of phenotypes and genotypes of round gobies (*Neogobius melanostomus*) used for this study.

Site	Region	Coordinates (Lat, Long in EPSG:4326)	Local salinity (PSU)	Salinities where sperm were tested (PSU)
Sozopol	ancestral Black Sea	42,4333, 27,7011	14-18	0; 8; 16; 24; 32
Kindvig	Southern Baltic	55,2788, 12,4463	~15	1; 5; 10; 15; 20; 25; 30
Karrebaeksminde	Southern Baltic	55,1747, 11,6409	~15	1; 5; 10; 15; 20; 25; 30
Guldborgsund	Southern Baltic	54,8558, 11,7497	8-22	0; 16; 32
Travemünde	Southern Baltic	53,8956, 10,7979	10-15	0; 16
Mariehamn	Northern Baltic	60,1012, 19,9227	~5	1; 5; 10; 15; 20; 25; 30
Turku	Northern Baltic	60,4209, 22,0915	~5	1; 5; 10; 15; 20; 25; 30
Raahe	Northern Baltic	64,6586, 24,4158	~2	1; 5; 10; 15; 20; 25; 30
Elbe	western European rivers	53,5491, 9,9861	0	0; 16; 32
Rhine	western European rivers	51,8609, 6,0715	0	0; 16; 32
Danube	ancestral river	44,0538, 26,6171	0	0; 8; 16; 24; 32

Supplementary table 1 cont.

Site	Genotpyes sequenced	Genotypes kept after filtering	Filtered genotypes sampled together with sperm	Library	Sequencing year	Data from
Sozopol	N = 30	N = 29	N = 10	3, 4 & 5	2019	This study
Kindvig	N = 31	N = 5	N/A	3, 4 & 5	2019	Green, Havenhand et al. 2020
Karrebaeksminde	N = 20	N = 13	N = 5	3, 4 & 5	2019	Green, Havenhand et al. 2020
Guldborgsund	N = 32	N = 32	N = 19	1 & 2	2017	Green, Niemax et al. 2021
Travemünde	N = 31	N = 31	N/A	1 & 2	2017	Green, Niemax et al. 2020
Mariehamn	N = 30	N = 26	N = 8	3, 4 & 5	2019	Green, Havenhand et al. 2020
Turku	N = 30	N = 27	N = 4	3, 4 & 5	2019	Green, Havenhand et al. 2020
Raahe	N = 22	N = 22	N = 7	3, 4 & 5	2019	Green, Havenhand et al. 2020
Elbe	N = 30	N = 29	N = 5	1 & 2	2017	Green, Niemax et al. 2021
Rhine	N = 32	N = 32	N = 8	1 & 2	2017	Green, Niemax et al. 2021
Danube	N = 31	N = 18	N = 12	3, 4 & 5	2019	This study

Supplementary Table 2. Results from linear models of sperm performance in *Neogobius melanostomus* across 8 introduced sites from previously published data together with two ancestral (freshwater marked with asterix, brackish marked with cross) sites from this study. Models were performed independently for sperm velocity and sperm motility and compared performance in four different conditions: freshwater (**a**) (0-1 practical salinity units (PSU)); (**b**) brackish (15-16 PSU); (**c**) marine (30-32 PSU); (**d**) local (varying PSU, see table for details). Note also that estimates for Marine and Local models are offsett to show the estimate of the first site in the list. * = p < 0.05, ** = p < 0.01, *** = p < 0.001.

**a) Sperm velocity tested in freshwater (0-1 PSU)**								
**Model:**	**Num.df**	**Denom. df**	**S.S.**	**M.S.**	**F**	***p***	**R^2**	**Adj. R^2**
lm(Velocity ~ Site, data = sperm in 0-1 PSU)	10	86	4172.0	417.20	4.05	<0.001	0.320	0.241
**Site (group)**	**N**	**Estimate**	**S.E.**	**lower CI**	**upper CI**	***t***	***p***	**Sing.**
Danube* (Intercept)	14	62.89	2.713	57.50	68.29	23.18	*<0.001*	***
Rhine	5	-6.94	5.288	-17.46	3.57	-1.31	0.193	
Elbe	4	-12.68	5.755	-24.12	-1.24	-2.20	*0.030*	*
Raahe	7	-3.02	4.699	-12.36	6.33	-0.64	0.523	
Turku	5	-7.71	5.288	-18.23	2.80	-1.46	0.148	
Mariehamn	10	-10.17	4.203	-18.52	-1.81	-2.42	*0.018*	*
Travemünde	20	-9.71	3.537	-16.74	-2.68	-2.75	*0.007*	**
Guldborgsund	9	-18.34	4.337	-26.96	-9.72	-4.23	*<0.001*	***
Karrebaeksminde	6	-13.80	4.953	-23.65	-3.95	-2.79	*0.007*	**
Kindvig	6	-18.22	4.953	-28.07	-8.37	-3.68	*<0.001*	***
Sozopol†	11	-20.62	4.090	-28.75	-12.49	-5.04	*<0.001*	***
**b) Sperm motility tested in freshwater (0-1 PSU)**								
**Model:**	**Num.df**	**Denom. df**	**S.S.**	**M.S.**	**F**	***p***	**R^2**	**Adj. R^2**
lm(Motility ~ Site, data = sperm in 0-1 PSU)	10	86	0.482	0.048	5.31	<0.001	0.382	0.310
**Site (group)**	**N**	**Estimate**	**S.E.**	**lower CI**	**upper CI**	***t***	***p***	**Sing.**
Danube* (Intercept)	14	0.09	0.026	0.04	0.15	3.73	*<0.001*	***
Rhine	5	-0.01	0.049	-0.11	0.09	-0.24	0.809	
Elbe	4	-0.07	0.054	-0.17	0.04	-1.21	0.229	
Raahe	7	-0.08	0.044	-0.16	0.01	-1.70	0.092	
Turku	5	0.21	0.050	0.11	0.31	4.23	*<0.001*	***
Mariehamn	10	-0.01	0.040	-0.09	0.07	-0.20	0.844	
Travemünde	20	-0.04	0.033	-0.10	0.03	-1.11	0.269	
Guldborgsund	9	-0.09	0.041	-0.17	-0.01	-2.19	*0.032*	*
Karrebaeksminde	6	0.10	0.047	0.01	0.19	2.15	*0.034*	*
Kindvig	6	0.01	0.047	-0.08	0.10	0.22	0.823	

Sozopol†	11	-0.08	0.038	-0.16	-0.00	-2.10	*0.038*	*
**c) Sperm velocity tested in brackish conditions (15-16 PSU)**								
**Model:**	**Num.df**	**Denom. df**	**S.S.**	**M.S.**	**F**	***p***	**R²**	**Adj. R²**
lm(Velocity ~ Site, data = sperm in 15-16 PSU)	10	87	64610	6461	15.46	<0.001	0.640	0.598
**Site (group)**	**N**	**Estimate**	**S.E.**	**lower CI**	**upper CI**	***t***	***p***	**Sing.**
Sozopol† (Intercept)	12	102.87	5.902	91.14	114.60	17.43	*<0.001*	***
Kindvig	6	7.30	10.222	-13.02	27.61	0.71	0.477	
Karrebaeksminde	6	3.98	10.222	-16.34	24.30	0.39	0.698	
Guldborgsund	9	4.68	9.015	-13.23	22.60	0.52	0.605	
Travemünde	20	18.42	7.465	3.58	33.25	2.47	*0.016*	*
Mariehamn	10	-15.03	8.754	-32.43	2.37	-1.72	0.089	
Turku	5	12.51	10.882	-9.12	34.14	1.15	0.254	
Raahe	7	-6.13	9.723	-25.45	13.20	-0.63	0.530	
Elbe	4	-30.81	11.804	-54.27	-7.35	-2.61	*0.011*	*
Rhine	5	-25.00	10.882	-46.63	-3.37	-2.30	*0.024*	*
Danube*	14	-61.47	8.043	-77.46	-45.49	-7.64	*<0.001*	***
**d) Sperm motility tested in brackish conditions (15-16 PSU)**								
**Model:**	**Num.df**	**Denom. df**	**S.S.**	**M.S.**	**F**	***p***	**R²**	**Adj. R²**
lm(Motility ~ Site, data = sperm in 15-16 PSU)	10	87	1.598	0.160	22.25	<0.001	0.719	0.687
**Site (group)**	**N**	**Estimate**	**S.E.**	**lower CI**	**upper CI**	***t***	***p***	**Sing.**
Sozopol† (Intercept)	12	0.13	0.025	0.08	0.17	5.12	*<0.001*	***
Kindvig	6	0.41	0.042	0.33	0.50	9.78	*<0.001*	***
Karrebaeksminde	6	0.21	0.042	0.12	0.29	4.92	*<0.001*	***
Guldborgsund	9	0.02	0.037	-0.06	0.09	0.49	0.623	
Travemünde	20	0.06	0.031	-0.00	0.12	1.92	0.058	
Mariehamn	10	0.05	0.036	-0.02	0.13	1.48	0.143	
Turku	5	0.04	0.045	-0.05	0.13	0.95	0.345	
Raahe	7	-0.05	0.040	-0.13	0.03	-1.22	0.237	
Elbe	4	-0.08	0.049	-0.18	0.02	-1.63	0.106	
Rhine	5	-0.10	0.045	-0.19	-0.01	-2.12	*0.037*	*
Danube*	14	-0.12	0.033	-0.19	-0.05	-3.59	*0.001*	***
**e) Sperm velocity tested in marine conditions (30-32 PSU)**								
**Model:**	**Num.df**	**Denom. df**	**S.S.**	**M.S.**	**F**	***p***	**R²**	**Adj. R²**
lm(Velocity ~ Site, offset = global mean, data = sperm in 30-32 PSU)	9	65	11224	1247.06	5.27	<0.001	0.422	0.342
**Site (group)**	**N**	**Estimate**	**S.E.**	**lower CI**	**upper CI**	***t***	***p***	**Sing.**
Sozopol† (Intercept)	11	6.89	4.638	-2.37	16.16	1.49	0.142	
Kindvig	6	8.45	7.807	-7.15	24.04	1.08	0.283	
Karrebaeksminde	6	-5.42	7.807	-21.01	10.18	-0.69	0.490	
Guldborgsund	9	11.44	6.914	-2.37	25.24	1.65	0.103	

Mariehamn	9	-10.81	6.914	-24.62	3.00	-1.56	0.123	
Turku	5	-5.00	8.297	-21.57	11.57	-0.60	0.549	
Raahe	6	-7.91	7.807	-23.51	7.68	-1.01	0.314	
Elbe	4	-7.22	8.981	-25.16	10.71	-0.80	0.422	
Rhine	5	-10.53	8.297	-27.10	6.04	-1.27	0.209	
Danube*	14	-27.64	6.198	-40.01	-15.26	-4.46	*<0.001*	***
**f) Sperm motility tested in marine conditions (30-32 PSU)**								
**Model:**	**Num.df**	**Denom. df**	**S.S.**	**M.S.**	**F**	***p***	**R²**	**Adj. R²**
lm(Motility ~ Site, offset = global mean, data = sperm in 30-32 PSU)	9	65	0.070	0.008	2.01	0.052	0.218	0.110
**Site (group)**	**N**	**Estimate**	**S.E.**	**lower CI**	**upper CI**	***t***	***p***	**Sing.**
Sozopol† (Intercept)	11	-0.01	0.019	-0.05	0.03	-0.61	0.544	
Kindvig	6	0.09	0.032	0.02	0.15	2.71	*0.009*	**
Karrebaeksminde	6	0.02	0.032	-0.04	0.08	0.68	0.500	
Guldborgsund	9	0.04	0.028	-0.01	0.10	1.55	0.125	
Mariehamn	9	0.01	0.028	-0.05	0.06	0.21	0.831	
Turku	5	0.04	0.036	-0.03	0.10	1.10	0.274	
Raahe	6	0.00	0.032	-0.06	0.07	0.09	0.929	
Elbe	4	-0.01	0.036	-0.08	0.07	-0.20	0.839	
Rhine	5	-0.01	0.034	-0.08	0.05	-0.43	0.665	
Danube*	14	-0.02	0.025	-0.07	0.03	-0.93	0.353	
**g) Sperm velocity tested in local conditions (see Site for values)**								
**Model:**	**Num.df**	**Denom. df**	**S.S.**	**M.S.**	**F**	***p***	**R²**	**Adj. R²**
lm(Velocity ~ Site, offset = global mean, data = sperm in local PSU)	10	87	55528	5552.8	18.24	<0.001	0.677	0.640
**Site (group)**	**N**	**Estimate**	**S.E.**	**lower CI**	**upper CI**	***t***	***p***	**Sing.**
Sozopol† (Intercept) (16 PSU)	12	8.63	5.037	-1.38	18.64	1.71	0.090	
Kindvig (15 PSU)	6	7.30	8.724	-10.04	24.64	0.84	0.405	
Karrebaeksminde (15 PSU)	6	3.98	8.724	-13.36	21.32	0.46	0.649	
Guldborgsund (16 PSU)	9	4.68	7.694	-10.61	19.98	0.61	0.544	
Travemünde (16 PSU)	20	18.42	6.371	5.75	31.08	2.89	*0.005*	**
Mariehamn (5 PSU)	10	-23.22	7.470	-38.07	-8.37	-3.11	*0.003*	**
Turku (5 PSU)	5	16.79	9.287	-1.67	35.25	1.81	0.074	
Raahe (5 PSU)	7	-24.39	8.298	-40.88	-7.89	-2.94	*0.004*	**
Elbe (0 PSU)	4	-52.65	10.073	-72.67	-32.63	-5.23	*<0.001*	***
Rhine (0 PSU)	5	-46.92	9.287	-65.38	-28.46	-5.05	*<0.001*	***
Danube* (0 PSU)	14	-39.97	6.864	-53.62	-26.33	-5.82	*<0.001*	***
**h) Sperm motility tested in local conditions (see Site for values)**								
**Model:**	**Num.df**	**Denom. df**	**S.S.**	**M.S.**	**F**	***p***	**R²**	**Adj. R²**
lm(Motility ~ Site, offset = global mean, data = sperm in local PSU)	10	87	1.747	0.175	16.956	<0.001	0.661	0.622

**Site (group)**	**N**	**Estimate**	**S.E.**	**lower CI**	**upper CI**	***t***	***p***	**Sing.**
Sozopol† (Intercept) (16 PSU)	12	-0.08	0.029	-0.14	-0.02	-2.78	*0.007*	**
Kindvig (15 PSU)	6	0.41	0.051	0.31	0.52	8.16	*<0.001*	***
Karrebaeksminde (15 PSU)	6	0.21	0.051	0.11	0.31	4.11	*<0.001*	***
Guldborgsund (16 PSU)	9	0.02	0.045	-0.07	0.11	0.41	0.682	
Travemünde (16 PSU)	20	0.06	0.037	-0.01	0.13	1.60	0.113	
Mariehamn (5 PSU)	10	0.24	0.044	0.16	0.33	5.57	*<0.001*	***
Turku (5 PSU)	5	0.28	0.054	0.17	0.38	5.13	*<0.001*	***
Raahe (5 PSU)	7	0.01	0.048	-0.08	0.11	0.28	0.782	
Elbe (0 PSU)	4	-0.10	0.059	-0.21	0.02	-1.63	0.106	
Rhine (0 PSU)	5	-0.04	0.054	-0.15	0.07	-0.78	0.435	
Danube* (0 PSU)	14	-0.03	0.040	-0.11	0.05	-0.76	0.449	

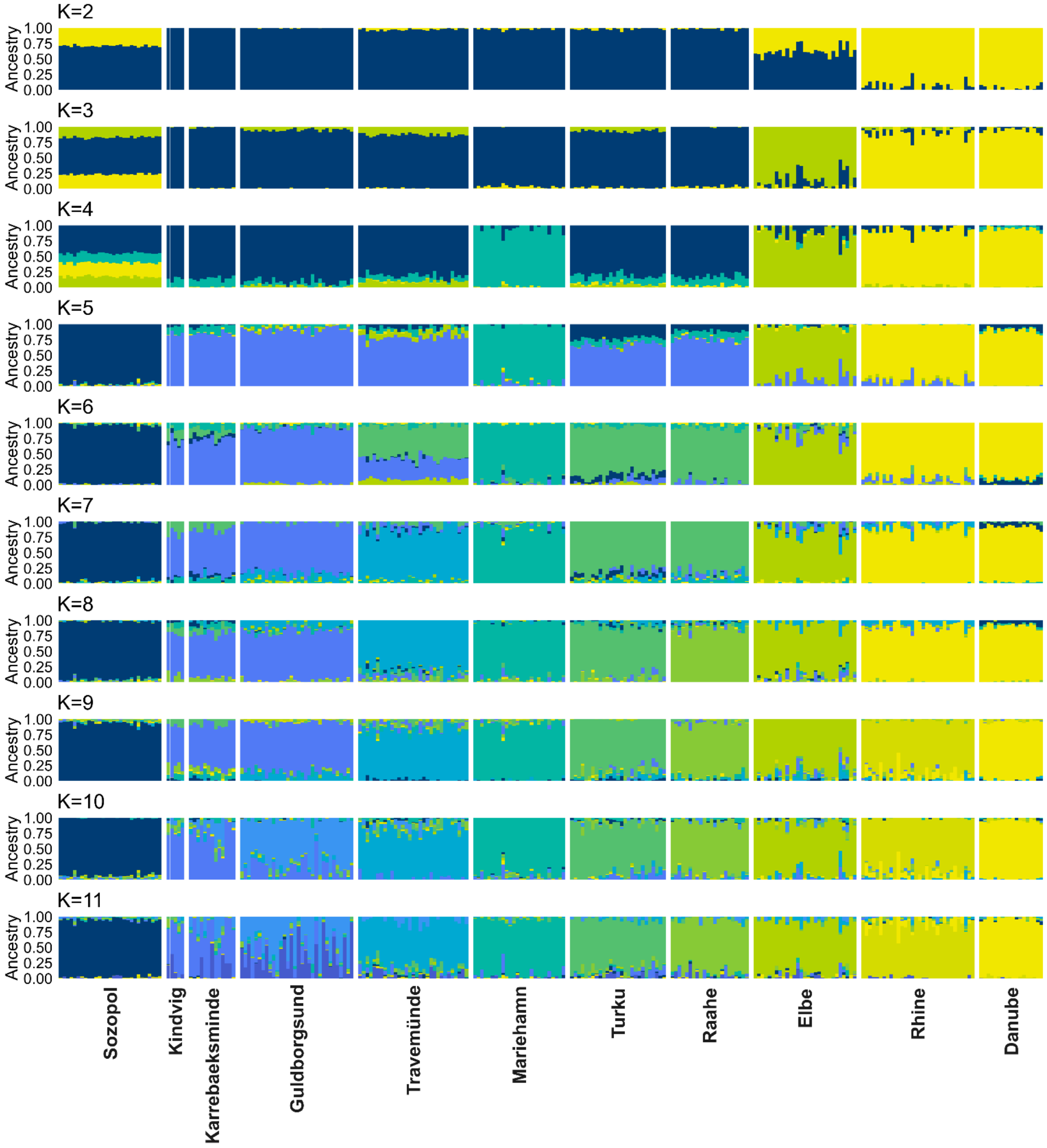

Supplementary Figure S1. Individual ancestry estimates using sNMF of 264 round gobies (*Neogobius melanostomus*) based on 13847 SNPs, for K = 2 - 11. Each vertical bar represents one individual and the colour the proportion of that individual assigned to the different K clusters.

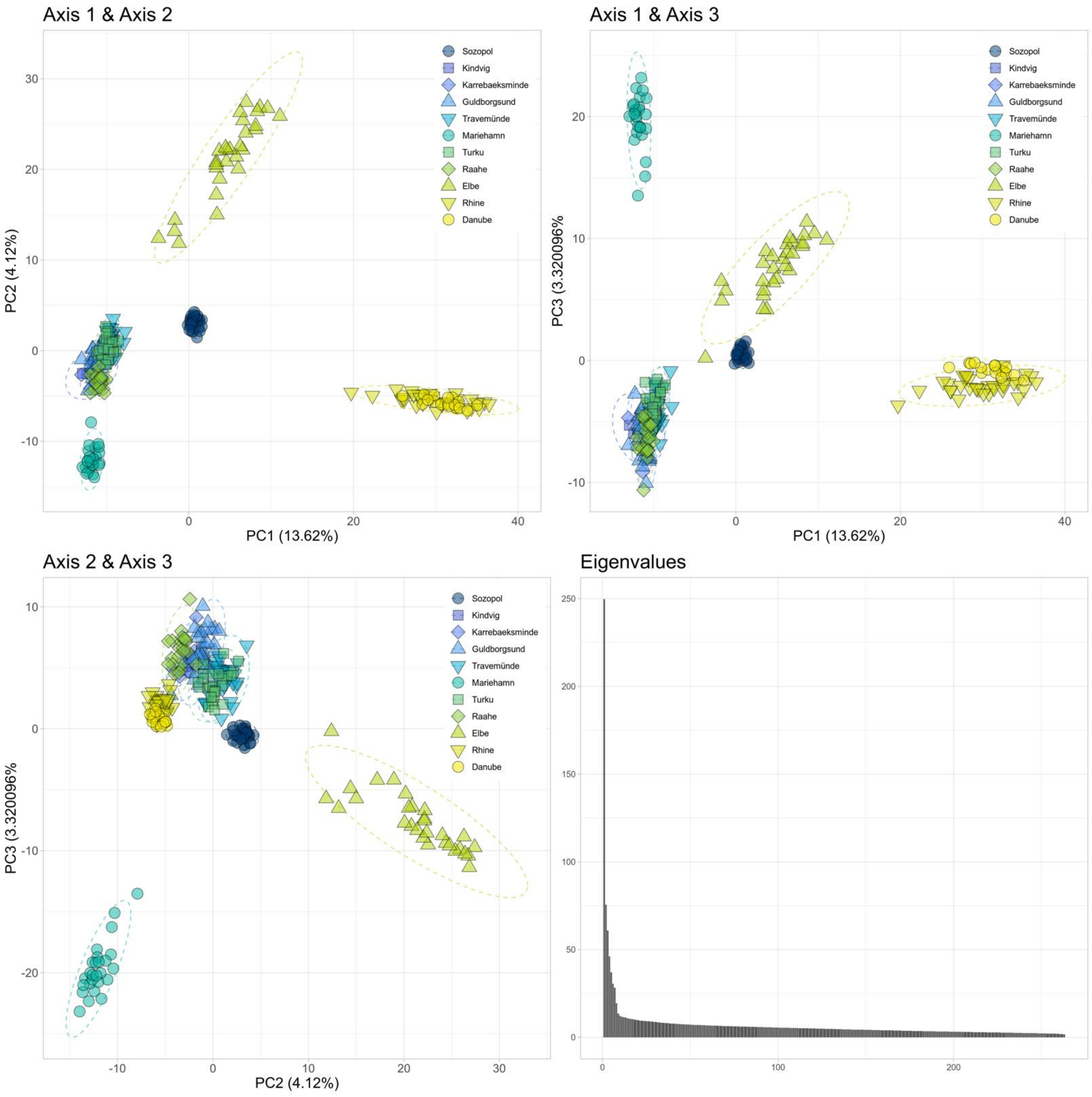

Supplementary Figure S2. Complete visualisation of axes 1-3 First components of a principal component analysis (PCA) on 264 round gobies (*Neogobius melanostomus*) calculated from 13847 SNPs. Name of the PCA panels shows the order components as x-axis and y-axis. Percentage of variation per axis is noted at each axis. Each point represents one individual, colours represent sampling origin and shape is used for better distinction between sites. Eigenvalues show the relative explanatory power of each component (axis).
